# Supplementary material for: Evaluating sequence and structural similarity metrics for predicting shared paralog functions
Source: NAR Genom Bioinform. 2025 Apr 26;7(2):lqaf051. doi: 10.1093/nargab/lqaf051 (PMC12034104; doi:10.1093/nargab/lqaf051)
Supplement: lqaf051_Supplemental_File [file lqaf051_supplemental_file.pdf]

# SUPPLEMENTARY DATA from:

# Evaluating Sequence and Structural Similarity Metrics for Predicting Shared Paralog Functions


**AUTHORS**

Olivier Dennler[1,2,3]* and Colm J. Ryan[1,2,3]*

[1] School of Medicine, University College Dublin, Dublin 4, Ireland
[2] School of Computer Science, University College Dublin, Dublin 4, Ireland
[3] Conway Institute, University College Dublin, Dublin 4, Ireland

* To whom correspondence should be addressed. Olivier Dennler, Email: olivier.dennler@ucd.ie, Colm J. Ryan, Email: colm.ryan@ucd.ie

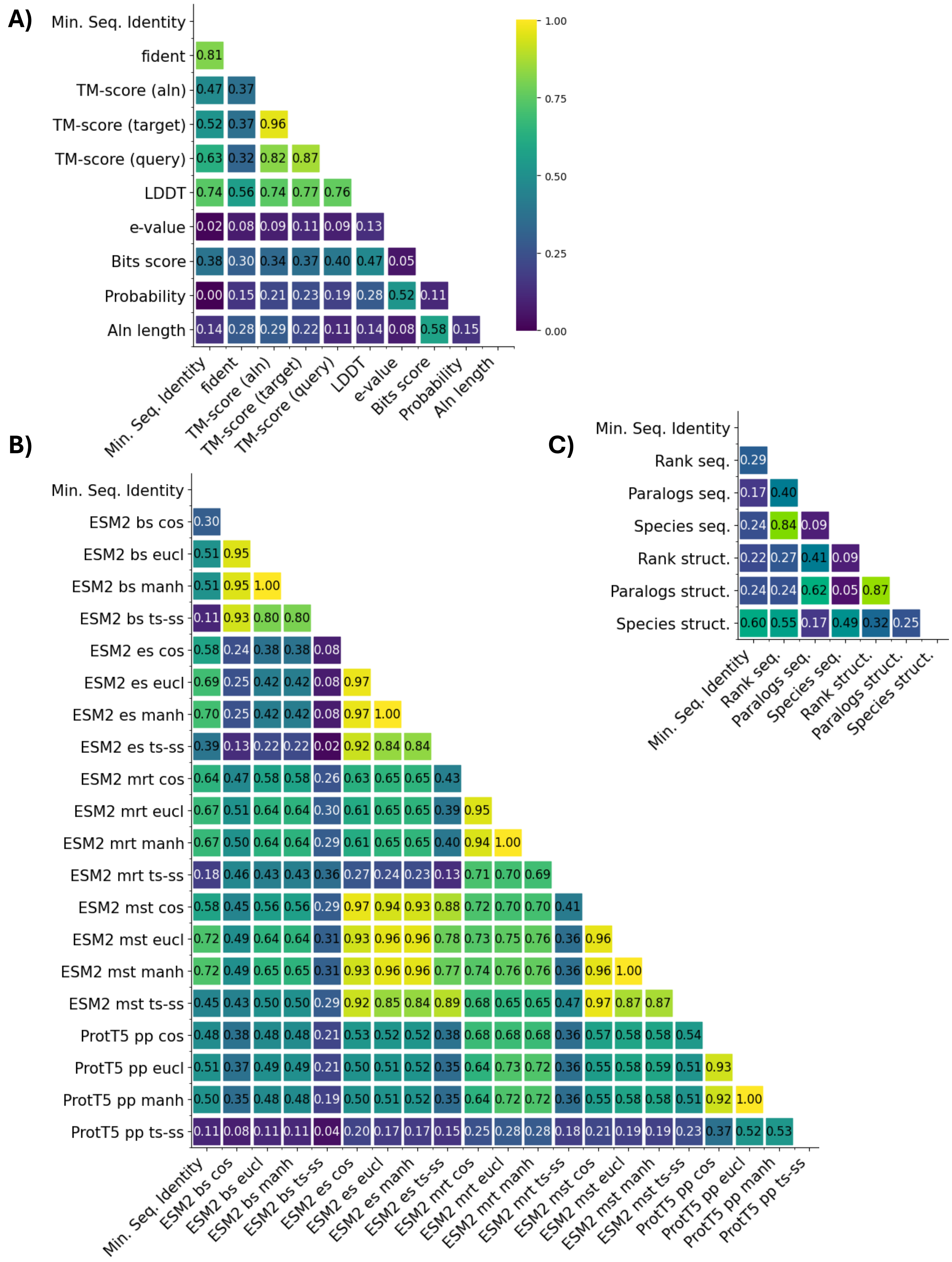


**Supplementary Fig. 1 | Pearson correlation coefficients (absolute values) between sequence features in 5,673 yeast paralog pairs. A)** For the nine predicted structure similarity features and sequence identity. **B)** For the twenty Protein Language Model

embedding features and sequence identity. **C)** For the six similarity search features and sequence identity.

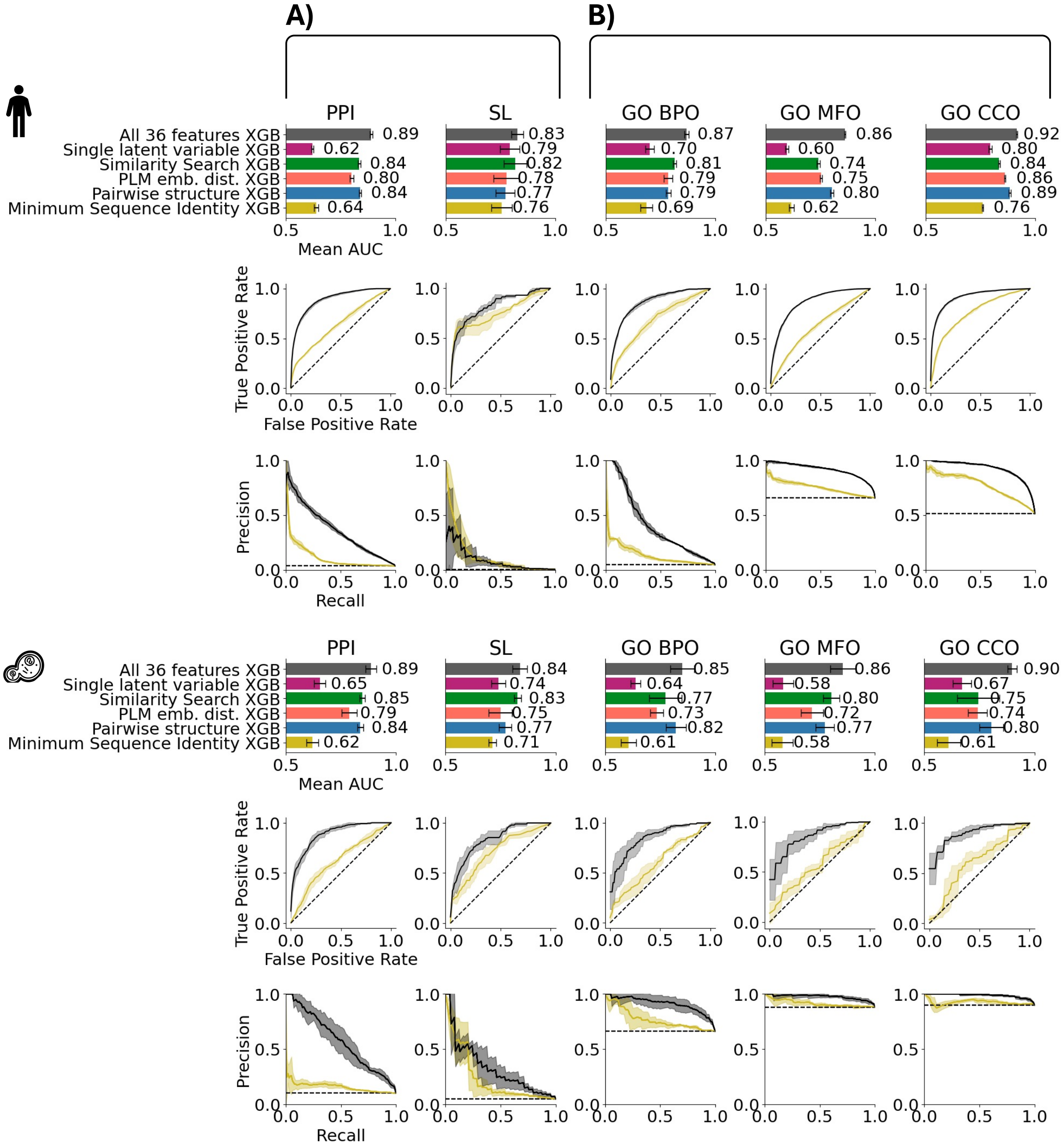


**Supplementary Fig. 2 | Integrating all features improves prediction of shared PPIs, synthetic lethality, and GO semantic similarity in human and in yeast.** Performances of an XGBoost classifier using all 36 sequence similarity features together (in grey/black) compared to a classifier using solely sequence identity (in yellow), the classifier using the nine predicted structure similarity features plus sequence identity (in blue), the classifier using the 20 PLM features plus sequence identity (in dark orange), the classifier using the six similarity search features plus sequence identity, and a classifier using only a single latent

variable representing all 36 features (in purple) for 4-fold cross-validation on shared functions prediction across five human datasets (PPI ; SL ; GO BPO ; GO MFO ; GO CCO) and their equivalent in yeast. Top chart shows the mean AUC on each dataset, ROC and precision-recall curves for each human datasets. Bottom chart shows the mean AUC on each dataset, ROC and precision-recall curves for each yeast datasets.

| | Bioplex | | Biogrid Phys. | | Biogrid Y2H | |
|---|---|---|---|---|---|---|
| | FET | Jaccard | FET | Jaccard | FET | Jaccard |
| "Functionally Redundant" Pairs | 3 515 | 421 | 17 831 | 45 431 | 2 910 | 234 |
| "Not Functionally Redundant" Pairs | 92 775 | 99 418 | 63 626 | 103 795 | 94 499 | 100 918 |
| Ratio | 3.6 % | 0.4 % | 21.9 % | 43.8 % | 3.0 % | 0.2 % |

**Supplementary Table 1 | Human Protein-Protein Interaction (PPI) datasets.** Table showing the number of paralog pairs classified as "shared function" based on PPI annotations in human. We used three sources of PPI data: Bioplex, Biogrid physical interactions (Phys.), Biogrid double hybrid only (Y2H). Then, "shared function" is defined using either thresholds on Fisher's exact test (FET) log p-value or thresholds on Jaccard index, yielding a total of 6 human PPI datasets overall. Note that the Bioplex dataset is the one used in the main manuscript as "Human PPI".

| | Social Inter. | | Biogrid Phys. | | Biogrid Y2H | |
|---|---|---|---|---|---|---|
| | FET | Jaccard | FET | Jaccard | FET | Jaccard |
| "Functionally Redundant" Pairs | 388 | 202 | 2 128 | 222 | 178 | 89 |
| "Not Functionally Redundant" Pairs | 3 501 | 3 636 | 779 | 2 329 | 3 582 | 3 731 |
| Ratio | 10.0 % | 5.2 % | 73.2 % | 8.7 % | 4.7 % | 2.3 % |

**Supplementary Table 2 | Yeast Protein-Protein Interaction (PPI) datasets.** Table showing the number of paralog pairs classified as "shared function" based on PPI annotations in

budding yeast. We used three sources of PPI data: Social Interactome from Michaelis et al. (2019), Biogrid physical interactions (Phys.), Biogrid double hybrid only (Y2H). Then, "shared function" is defined using either thresholds on Fisher's exact test (FET) log p-value or thresholds on Jaccard index, yielding a total of 6 yeast PPI datasets overall. Note that the Social Interactome dataset is the one used in the main manuscript as "Yeast PPI".

| | **Human SL** | | **Yeast SL** | |
|---|---|---|---|---|
| | **DepMap** | **Lenient** | **Neg. GI** ( $\varepsilon \leq -0.08$) | **SL** ( $\varepsilon \leq -0.35$) |
| **"Functionally Redundant" Pairs** | **116** | **403** | **218** | **96** |
| **"Not Functionally Redundant" Pairs** | **23 618** | **4 913** | **1 790** | **1 912** |
| **Ratio** | **0.5 %** | **7.6 %** | **10.9 %** | **4.8 %** |

**Supplementary Table 3 | Synthetic Lethality (SL) datasets.** Table showing the number of paralog pairs classified as "shared function" based on SL annotations in two species (human and budding yeast). For human, we used two definitions: a DepMap-based approach using CRISPR cancer cell line data, and a "lenient" approach that labels pairs as SL if they were detected at least once across multiple double-knockout (dKO) screens. For budding yeast, we applied two thresholds on SGA NxN screen: negative genetic interactions (Neg. GI - fitness ⩽ －0.08) and "SL" (fitness ⩽ －0.35). Consequently, each species has two SL datasets, giving four SL/Neg. GI datasets in total. Note that the DeMap dataset is the one used in the main manuscript as "Human SL", whereas "Yeast SL" is defined by applying the -0.35 threshold.

| | Human GO | | | Yeast GO | | |
|---|---|---|---|---|---|---|
| | BPO | MFO | CCO | BPO | MFO | CCO |
| **"Functionally Redundant" Pairs** | **967** | **9 382** | **5 035** | **336** | **495** | **513** |
| **"Not Functionally Redundant" Pairs** | **19 075** | **4 898** | **4 765** | **172** | **67** | **57** |
| **Ratio** | **4.8 %** | **65.7 %** | **51.4 %** | **66.1 %** | **88.1 %** | **90 %** |

**Supplementary Table 4 | Gene Ontology (GO) datasets.** Table showing the number of paralog pairs classified as "shared function" based on GO annotations in two species (human and budding yeast). For each species, three datasets are defined—BPO (biological process), MFO (molecular function), and CCO (cellular component)—based on the semantic distance between all GO terms (within that category) annotated for one gene and all terms for its paralog. Consequently, each species has three GO datasets, yielding a total of six GO datasets overall.

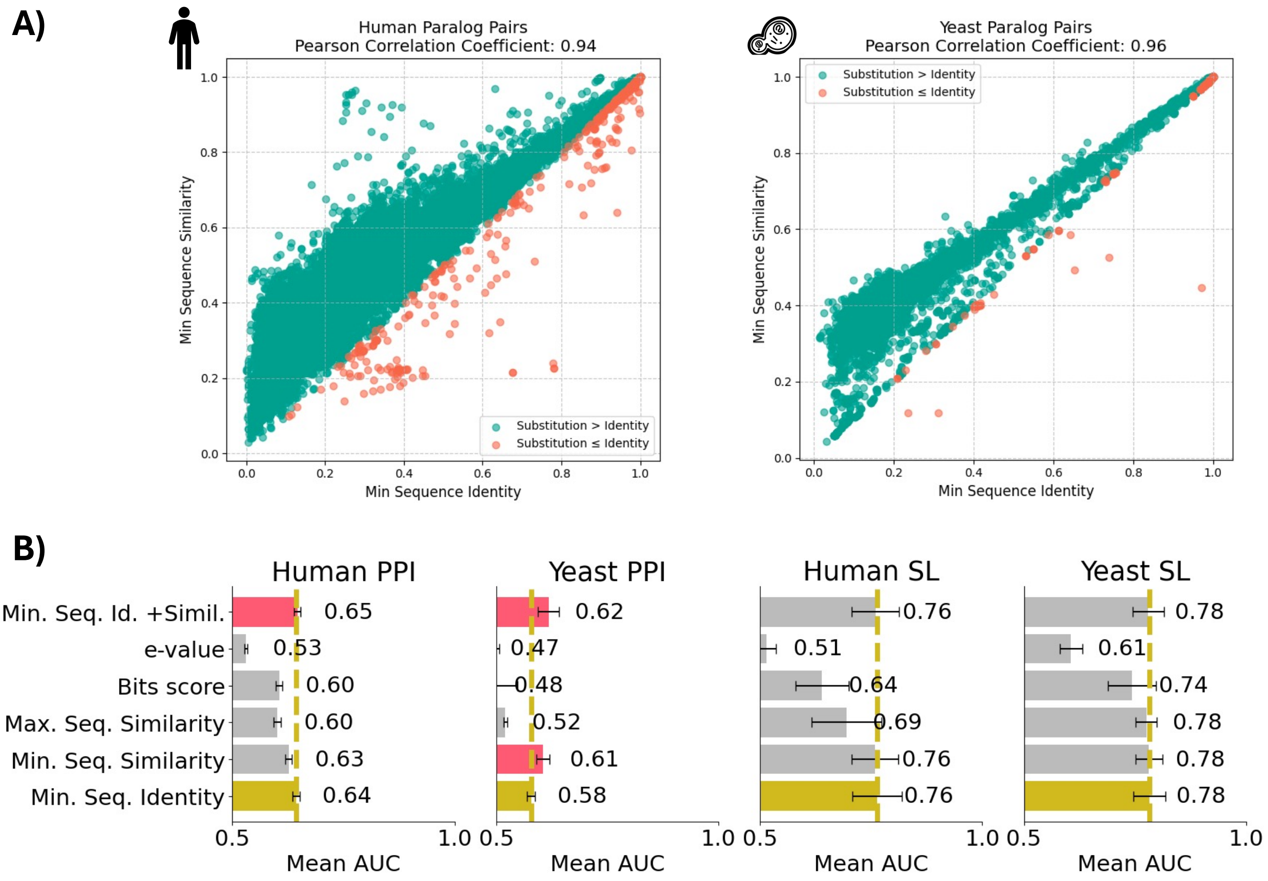


**Supplementary Fig. 3 | Sequence similarity features are highly redundant with sequence identity for capturing shared paralog function.** To assess whether we should consider using sequence similarity instead of sequence identity, we examined four different sequence-similarity-based features, all derived from a substitution matrix: minimum sequence similarity percentage, maximum sequence similarity percentage, MMseqs2 bits score, and MMseqs2 e-value. A) Correlations between sequence identity (from Ensembl database) and sequence similarity (positive matches in global pairwise alignment using a BLOSUM62 matrix) in human and yeast paralog pairs, each dot representing a paralog pair. B) Mean AUC values comparing these four individual sequence similarity features with minimum sequence identity (in yellow) for predicting shared functions across the four main datasets using four-fold cross-validation. We also considered a logistic regression classifier combining both minimum sequence identity and minimum sequence similarity ("Min. Seq. Id. + Simil."). Features with a mean AUC greater than the mean AUC of sequence identity are shown in red. Error bars show the standard deviation of AUC values from cross-validation.

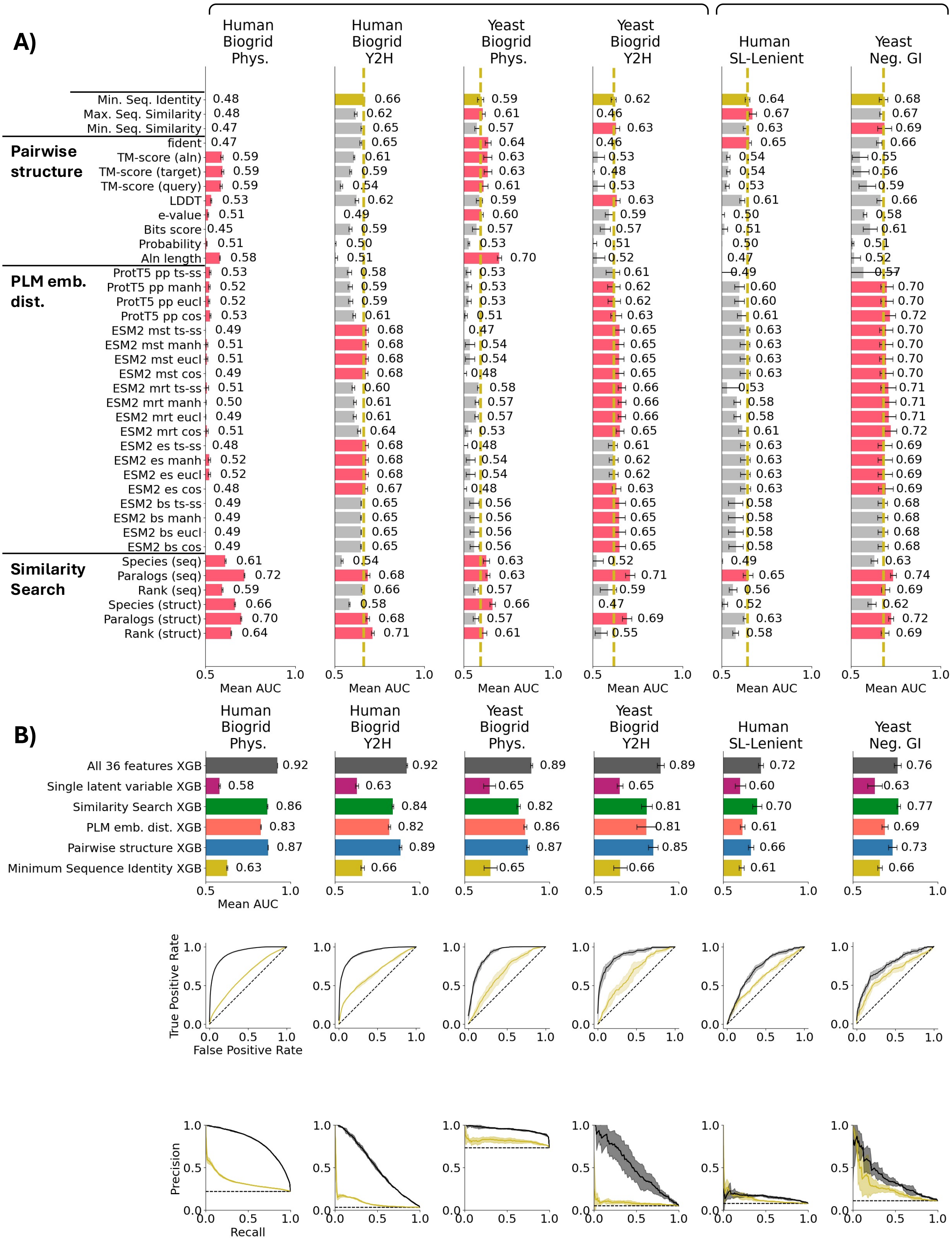

**Supplementary Fig. 4 | Validation across additional PPI/SL datasets demonstrates combined-features robustness.** A) Mean AUC values comparing all individual sequence features (sequence similarities, pairwise structure metrics, PLM embedding distances, and

similarity searches) with minimum sequence identity (in yellow) for predicting shared functions across six datasets using four-fold cross-validation. Features with a mean AUC higher than that of sequence identity appear in red. Error bars represent the standard deviation of AUC values from cross-validation. B) Performance of an XGBoost classifier using all 36 sequence similarity features (in grey/black) compared to a classifier using only sequence identity (in yellow), one using the nine predicted structure similarity features plus identity (in blue), one using the 20 PLM features plus identity (in dark orange), one using the six similarity search features plus identity, and a classifier using a single latent variable representing all 36 features (in purple). We evaluated by four-fold cross-validation on shared-function prediction across six additional datasets: PPI Biogrid physical (human), PPI Biogrid double hybrid only (human), PPI Biogrid physical (yeast), PPI Biogrid double hybrid only (yeast), SL "lenient" in dKO screens (human), and negative genetic interactions (Neg. GI) from yeast (fitness ≤ −0.35). The chart shows mean AUC, ROC, and precision–recall curves for the six additional datasets.

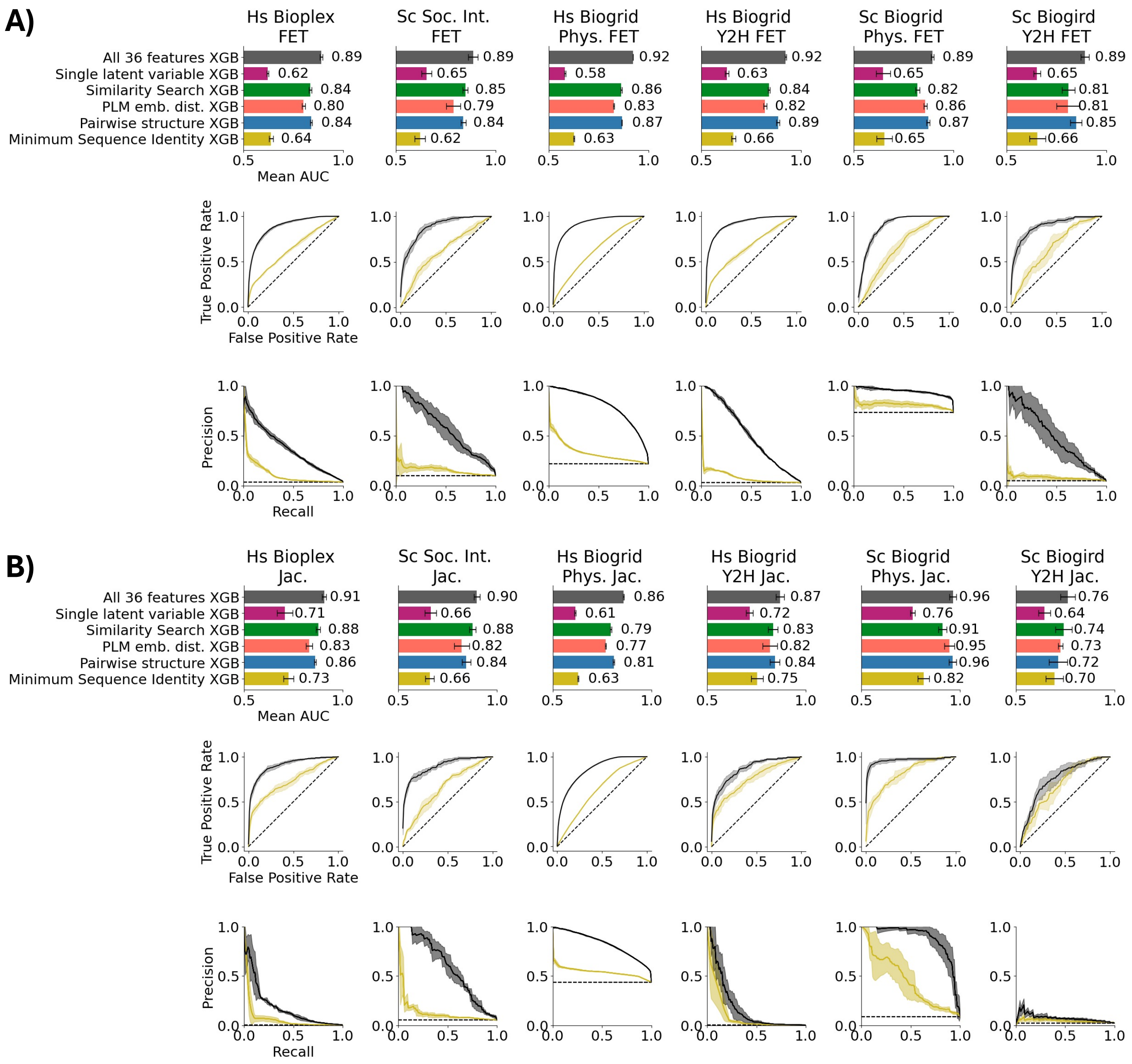


**Supplementary Fig. 5 | Combined features perform equally well on PPI datasets defined by FET or Jaccard thresholds.** We compared an XGBoost classifier using all 36 sequence similarity features (grey/black) to several alternatives: only sequence identity (yellow), the nine predicted-structure features plus identity (blue), the 20 PLM features plus identity (dark orange), the six similarity-search features plus identity, and a single latent variable representing all 36 features (purple). Using four-fold cross-validation, we evaluated shared-function prediction across two versions of each PPI dataset: one defined by Fisher's exact test (FET) thresholds (corresponding to the ones used in the main manuscript), and the other by Jaccard index thresholds. A) Top chart: mean AUC, ROC curves, and precision–recall curves for PPI datasets defined by FET thresholds. B) Bottom chart: the same evaluation for the PPI datasets but defined by Jaccard index thresholds.

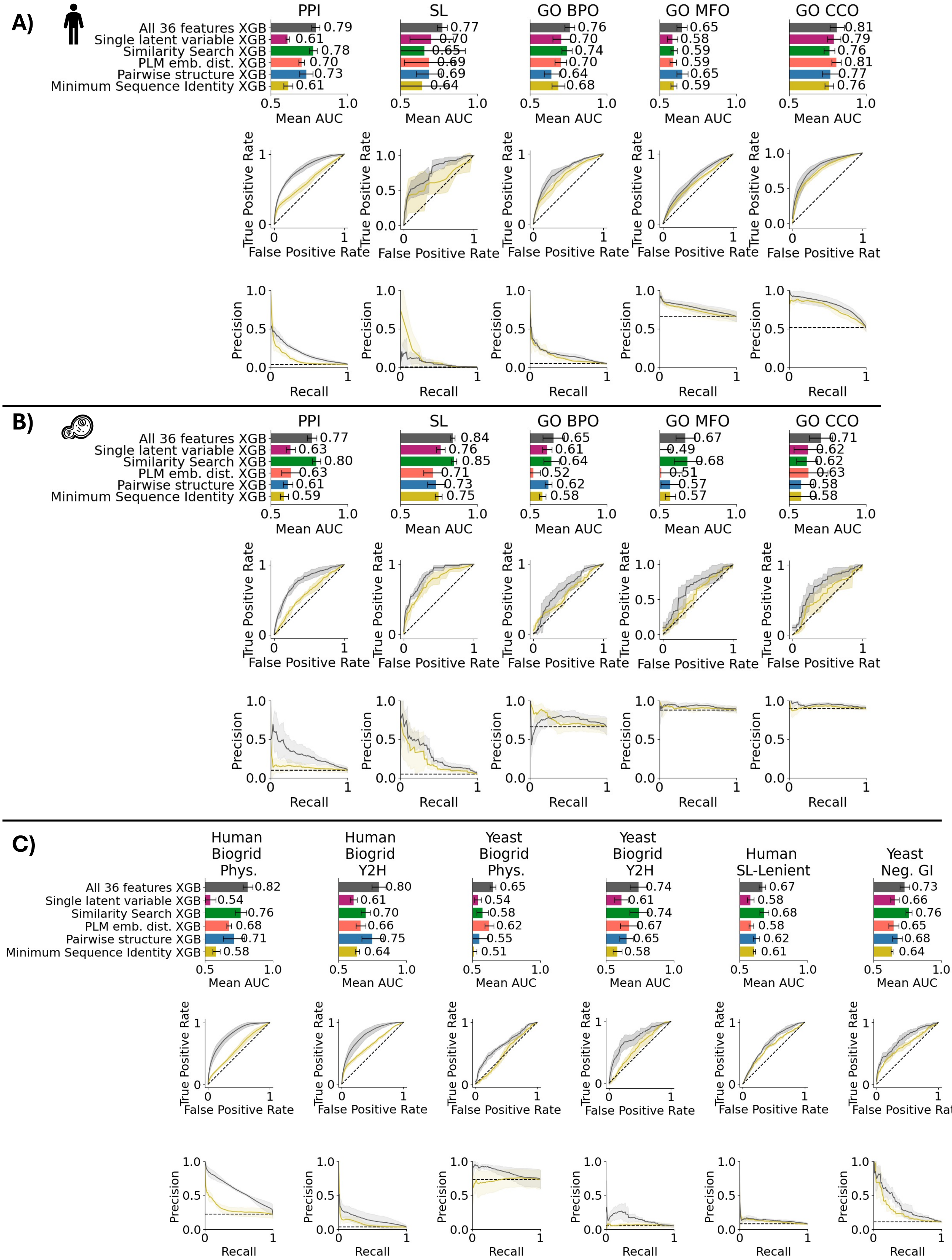


**Supplementary Fig. 6 | Integrating all features improves prediction even when predicting on unseen families.** We compared an XGBoost classifier using all 36 sequence

similarity features (grey/black) against several alternatives: only sequence identity (yellow), the nine predicted-structure features plus identity (blue), the 20 PLM features plus identity (dark orange), the six similarity-search features plus identity, and a single latent variable combining all 36 (purple). We performed family-split four-fold cross-validation, ensuring that training and test sets contain entirely different families, and assessed shared-function prediction across all datasets (Supplementary Tables 1–4). A) Top chart: Mean AUC, ROC curves, and precision–recall curves for the main human datasets. B) Middle chart: the same evaluation for the main yeast datasets. C) Bottom chart: the same evaluation for the additional datasets.
